# Choosing Death Over Survival: A Need to Identify Evolutionary Mechanisms Underlying Human Suicide

**DOI:** 10.3389/fpsyg.2021.689022

**Published:** 2021-11-03

**Authors:** Diya Chatterjee, Rishabh Rai

**Affiliations:** Department of Humanities and Social Sciences, Indian Institute of Technology, Kharagpur, India

**Keywords:** suicide, suicidology, proximate explanations, ultimate explanations, evolutionary psychology

## Abstract

The act of killing self contradicts the central purpose of human evolution, that is, survival and propagation of one’s genetic material. Yet, it continues to be one of the leading causes of human death. A handful of theories in the realm of evolutionary psychology have attempted to explain human suicide. The current article analyses the major components of certain prominent viewpoints, namely, Inclusive fitness, Bargaining model, Pain-Brain model, Psychological aposematism, and few other perspectives. The article argues that relatively more weightage has been given to understanding ultimate (the “why”) rather than proximate (the “how”) functionality of suicidal acts. Evolutionary theorists have consistently pointed out that to comprehensively understand a trait or behavior, one needs to delineate not only how it supports survival but also the evolution of the mechanisms underlying the trait or behavior. Existing theories on suicide have primarily focused on its fitness benefits on surviving kin instead of providing evolutionary explanations of the more complex mechanisms leading up to such self-destructive motivations. Thus, the current paper attempts to highlight this gap in theorizing while suggesting probable proximate explanations of suicide which stresses the need to diffuse attention paid to fitness consequences of the act alone. We speculate that such explorations are needed in order to build a robust and comprehensive evolutionary theory of human suicide.

## Introduction

The capacity to deliberately take one’s own life is perhaps one of the most distinctive acts that sets the human race apart. Even though other animals exhibit self-destructive behaviors, these acts have been attributed to objective factors. Such factors may range from species-typical self-sacrificial behavior, for instance, in honey bee species (*T. hyalinata*) for the protection of their nest (Shackleton et al., 2015, p. 1) to the apparent “adaptive suicide” behavior in leaf-cutting ants (*Atta cephalotes*) to eliminate the risk of propagating an established parasite to its kin (Trail, 1980, p. 81–82). These seemingly self-destructive behaviors seen in non-human species have mostly been fit into the evolutionary explanation of kin selection, whereby organisms sacrifice their own lives to ensure closest kin’s survival and reproductive success (Humphreys and Ruxton, 2019, p. 2). However, conventional biological explanations might not suffice when it comes to understanding self-destructive motivations in cognitively and socially enriched organisms, such as humans. According to a recent review of internationally applicable definitions for suicidal behaviors, suicide can be defined as an individual act whose outcome is death or life-threatening state, may be driven by deliberate or unintentional motive to die (De Leo et al., 2021, p. 6–8). Suicide is the loss of valuable human life, causing a premature termination of resourceful and functional human years. Globally, crude suicide rates per 100,000 population are 10.6 for both sexes, 7.7 for females and 13.5 for males (“Crude Suicide Rates,” World Health Organization, 2018). With suicide accounting for 1.4% of all deaths worldwide, becoming the 15th leading cause of death (World Health Organization, 2014), it is imperative to understand what might underlie the human choice of death over life. The phenomenon of suicide has been comprehensively discussed from various theoretical viewpoints spanning existential conceptualizations (Roberts and Lamont, 2014), psychodynamic theories, sociological theory of Durkheim (1897, 1958), hopelessness theory by Beck et al. (1975, 1990), psychache theory of Shneidman (1993), escape theory of Baumeister (1990), emotion dysregulation theory of Linehan (1993), interpersonal-psychological theory of Joiner (2005), and biological perspectives (Selby et al., 2014). Few other psychological theories of suicide, namely, the Fluid Vulnerability Theory (Rudd, 2006), the Integrated Motivational Model of suicidal behavior (O’Connor, 2011; O’Connor and Kirtley, 2018), and the 3-step “ideation to activation” framework (Klonsky and May, 2015), have attempted to delineate several aspects of suicidal behaviors from risk factors to cognitive and behavioral vulnerabilities.

Aforementioned schools of thought have proposed treatment paradigms for suicide, focusing on psychosocial and neurochemical underpinnings. However, a comprehensive understanding of any human behavior cannot exclude evolutionary discussions. For an integrated conceptualization of behavioral pathologies, the evolution of the behavior needs to be traced. Suicide apparently contradicts the basic intent of all living organisms, which is, the maintenance of biological fitness and propagation of one’s own genetic material across generations (deCatanzaro, 1980). In other words, to survive. What then might explain behaviors that go against the rule of thumb of evolution? Though suicidal behaviors have been consistently explored in evolutionary psychology (deCatanzaro, 1980, 1981, 1984, 1986, 1991; Soper, 2018; Humphreys and Ruxton, 2019), there might be some missing pieces to this puzzle. The continued occurrence of death by suicide in the society demands rigorous questioning to expand the existing literature.

In an attempt to move in that direction, the current article firstly aims to summarize prominent theories reflected in the literature on evolution of suicide (Brown et al., 2009; Tanaka and Kinney, 2011; Aubin et al., 2013; Selby et al., 2014; Syme et al., 2016; Soper, 2019a). The authors attempt to conceptually analyze these theories which focus on the ultimate functionality of suicide. However, to understand a behavior fully, proximate mechanisms leading up to the act need to be studied. The authors contemplate that a discourse on proximate psychological mechanisms underlying suicide, woven into an evolutionary framework, might provide the missing pieces to this puzzling human act. This paper is an outcome of preliminary thought processes and hopes to stimulate further relevant hypotheses and research endeavors.

## The Evolutionary Understanding of Suicide

### A Summary of Existing Theories

#### Suicidal Behavior to Promote Inclusive Fitness

One of the first evolutionary explanations of suicidal behaviors was proposed by deCatanzaro (1986). deCatanzaro proposed a mathematically derived model which highlighted the likelihood of self-destructive behaviors to be a function of two organismic factors: an individual’s reproductive value and perceived degree of burdensomeness on genetic relatives (deCatanzaro, 1986; Brown et al., 2009, p. 1). According to the theory, self-destructive acts result from perceived burdensomeness and low reproductive potential of an individual, incurring large fitness costs on biological relatives. Hence, killing oneself would increase the availability of resources to surviving kin and thereby enhance their likelihood of survival and reproduction (deCatanzaro, 1980, p. 269–270; deCatanzaro, 1984, p. 82). deCatanzaro’s model was based on Hamilton’s (1964, 1970, 1971) evolutionary biological proposition of how self-sacrifice may have beneficial effects on survival of kin, a process referred to as “kin selection” (Hamilton, 1964, 1970, 1971). The model, thus, contends that individuals with some perceived infirmities, for example, persons with a debilitating illness, would be more likely to show self-destructive motivation by virtue of their reduced reproductive potential and increased sense of burdensomeness on close kin (Syme, 2017). Another condition triggering self-destructiveness might be age or senescence (deCatanzaro, 1991). However, neither illness nor age alone can lead a person to his death, but other factors, such as emotional state of the organism, kin solicitude, and parental care, can influence the probability of dying by suicide in socially complex species like human beings (deCatanzaro, 1991, p. 14–16, 20–21; deCatanzaro, 1995, p. 387).

#### The Bargaining Model of Suicidal Behavior

Systematic reviews of suicidal behaviors have reported that self-harm behavior, with or without suicidal motives, can serve as a significant predictor of eventual suicide (Quarshie et al., 2020, p. 1; Grandclerc et al., 2016, p. 1–2; Mars et al., 2019, p. 327–328). The Bargaining Model of suicidal behavior attempts to explain this reported transition of self-harm to suicide from an evolutionary standpoint. The model, originally proposed by anthropologist Edward Hagen (1999, 2003) as a model for depression, states that non-lethal self-injurious behavior (SIB) is an appeal by less fortunate individuals on more resourceful kin for gaining a desirable outcome, such as affection or sympathy, which in certain cases might result in lethal attempts or suicide (Hagen, 2003, p. 95; Stengel, 1952, p. 22–24; Stengel, 1956, p. 117–120; Syme et al., 2016, p. 180–181). It is empirically supported that repeated non-lethal self-harm increases the likelihood of future suicide (Ciuhodaru et al., 2012, p. 767; Geulayov et al., 2019, p. 1051). Multiple reports of non-lethal self-harm behavior eventually turning into cases of suicide also exist in the literature (Syme et al., 2016, p. 181). Hence, from the evolutionary point of view, the bargaining model explains suicide as “costly signals of need” whereby an individual, after encountering a perceived fitness threat (such as, being restricted from making a particular choice), resorts to a costly signal of self-harm (such as, wrist slashing), with the intent of communicating one’s need to the involved kin, which might lead to the removal of the fitness threat previously imposed by more resourceful others (i.e., the particular choice of the individual being accepted), provided he or she survives the non-lethal attempt (Syme, 2019, p. 1–3). According to this view, suicide attempts are like bargains and completed suicides are “accidental outcomes” of a risky game-like strategy to influence social partners (Syme et al., 2016, p. 181).

#### The Pain-Brain Hypothesis of Suicide

Experience of intense pain, physiological or psychological, is one of the most compelling of human experiences (Walters and Williams, 2019, p. 1). Pain plays an adaptive role by guiding organisms to perceive a fitness threat and move toward ending or escaping from it (Klein, 2015, see book overview). Human pain is a complex phenomenon which comprises sensory, emotional, cognitive, and social elements (Walters and Williams, 2019, p. 2). The discussion of pain in this text will concern psychological and social forms of pain. Human beings often seek to terminate psychological or social pain by resorting to suicidal behaviors that would end life and thereby end pain (Eisenberger, 2012; Soper, 2019a, p. 457–458). However, psychological pain cannot be the only explanatory variable for suicidal acts and this realization brings forth another relevant variable: the brain (Soper, 2019a, p. 458–459). The variable that makes suicide available exclusively to humans and not to other animals is the capacity of self-consciousness, and it is in adolescence that humans overcome a claimed “cognitive ‘floor’ for suicide” (Perry, 2014, p. 110). This cognitive floor is a sort of cognitive limitation that is said to exist in prepubescents or intellectually incapacitated individuals. Persons belonging to these two groups might thus lack the intellectual ability to contemplate conscious self-killing (Baechler, 1975/1979, p. 38). On the other hand, a human adult, who has adequate “cognitive sophistication,” can think in abstract terms and carry out the executive planning needed for deliberate self-killing (Soper, 2019a, p. 459). Thus, according to this hypothesis, conscious self-destruction is a function of two adaptive mechanisms which interact together, that is, the experience of psychological pain and the possession of cognitive sophistication, enabling the willful cessation of one’s own life. The pain-brain theory further elucidates the function of: (a) front-line pain-related and brain-related factors called “fenders” which serve as buffers against suicidal behavior, for example, self-deceptive defenses which maintain an emotional homeostasis and prevents the pain from reaching an extreme intolerability, and (b) last-line factors called “keepers” which work as “reactive, antisuicide, evolved psychological mechanisms,” for example, making sense of the pain or loss of psychomotor energy which might otherwise be needed to attempt any form of self-destructive behavior (Soper, 2018, p. 125–245). Together, these proposed conditions, which have been termed by Soper as “suigiston” in the “pain-brain” theory, can determine the likelihood of an individual attempting to take one’s own life (Soper, 2019b, p. 37).

#### Psychological Aposematism and Other Perspectives

Borrowing from evolutionary biology, the concept of Aposematism has been used by theorists to suggest a novel stance on human suicide (Wiley, 2020, p. 228–229). Aposematism is a term in ecology and evolution (Wallace, 1867; Poulton, 1890). It refers to a mechanism by which certain evolved conspicuous color patterns in certain animals work to attract predators after which a toxic and unpalatable substance is released by the animals to deter further hunting of their species by the predating organisms (Rowe and Guilford, 2000, p. 261). For example, wasp species *Aculeate hymenoptera* possess a bright yellow and black coloration, which attracts birds to prey on them, following which they release a potent venom that harm the vertebrates, thereby conditioning predators to avoid insects with similar coloration (Schmidt, 2008, p. 4076). Suicidologists have speculated that a sort of psychological aposematism may explain the adaptive nature of human suicide. In case an individual dies by suicide, the aposematic consequence is two-fold: (a) The negative impact of death would deter other at-risk individuals from engaging in self-destructive behavior and (b) allow community members to develop preventive measures for persons who might engage in suicidal behaviors (Wiley, 2020, p. 228). Since evolutionary traits are claimed to be hereditary (Darwin, 1909), the aposematic trait could also be inherited and the mechanism is conceived to benefit people with the same phenotype, thereby resulting in the sustenance of that trait owing to its net fitness benefits despite the initial death of an organism (Wiley, 2020, p. 227–228).

Another claim that discusses an apparently disadvantageous circumstance in actually preventing suicide has been furthered by certain theorists. This stance holds that human conditions of illness, psychiatric in particular, might in fact discourage a person from taking one’s own life owing to the presence of associated symptoms of sadness, pessimism, lethargy, and lack of motivation (Aubin et al., 2013, p. 6878–6,879; Nesse, 2000, p. 14, 16, 18). Such a contention points toward the adaptive nature of psychiatric illnesses and how the associated symptoms may be working as a dissuading factor against the physical and psychological effort involved in taking one’s own life.

Both the above-mentioned propositions view suicide as a heritable trait which may or may not be present in the genetic make-up of an individual. Somewhat different from this understanding is another contention that views the likelihood of suicide as universal and claims that all individuals have the same probability to destroy self and that this trait only gets expressed in some while remaining dormant in others (Szentes and Thomas, 2013, p. 427). The proposition further assumes that if an individual dies by suicide, reproductive resources initially taken up by him/her becomes available and gets redistributed uniformly across the population, thereby creating more fitness opportunities for others, irrespective of kinship to the deceased person (Szentes and Thomas, 2013, p. 427–428). This mathematically elaborated claim that suicide is a ubiquitous trait, which may or may not get expressed, and in case it gets expressed, the populations with suicides would grow and have fitness benefits, is a novel yet debatable perspective. Further scientific exploration is called for explaining this potent threat to human existence which entails severe costs in terms of psychological pain and bereavement for surviving kin.

### A Critique of Summarized Theories: Identifying the Need for Proximate Explanations

The above discussion brings forth the evolutionary understanding of suicide which mostly focuses on the ultimate function of the act. These theories have explained suicidal behaviors in terms of its benefit to surviving kin (for example, inclusive fitness theory) or as adaptive acts that remove the person from undesirable conditions of survival (for example, bargaining and pain-brain hypothesis). However, critically viewing these theories have raised certain issues which the paper attempts to emphasize in upcoming sections.

The position taken by deCatanzaro in his inclusive fitness theory of suicide has found support in his own and in the work of others (deCatanzaro, 1980, 1984; Syme, 2017). The theory fits comfortably into the first rule of thumb of evolution, that is, survival and furthering one’s own genetic material through reproduction. The theory suggests that an individual with low reproductive potential and perceived burdensome to kin is more likely to die by suicide, thereby creating reproductive opportunities and making other resources available to close kin. Self-sacrificial behavior for defending the colony in sterile members of the largest order of insects, Hymenoptera, has been widely noted (Shorter and Rueppell, 2012, p. 2–3). Global data from research in humans have shown that there is an overall increase in suicide rates after the age of 60years (Shah et al., 2016, p. 3–6), and suicidal older adults have been reported to experience lowest social support, lowest sense of belonging, and highest interpersonal difficulties (Harrison et al., 2010, p. 6–8). Among other potent risk factors for suicide in older adults, identified across various studies, reduced functional capacity owing to physical or psychiatric illness and lack of social connectedness with close kin appear to be most important (Conwell et al., 2011, p. 2–6). Syme et al. (2016, p. 189) and Syme (2017, p. 1–2) have reported that in resource scarce environments at higher latitudes, like the Arctics, suicide among non-reproductive and infirm kin who are incurring high fitness costs have found to support the inclusive fitness model of suicide. These evidences point toward the role of reproductive health and functionality along with interpersonal variables that determine the individual’s position within the social network in motivating self-destructive acts, thereby validating deCatanzaro’s claims on suicide. However, the likelihood of inclusive fitness theory in explaining all sorts of suicide might be low, for example, in individuals with no deficit in reproductive capacities or in cases where competition for resources may be adequately met by actions other than killing self (Szentes and Thomas, 2013, p. 426–427). Moreover, this theory explains the ultimate functionality of suicide as an outcome of selection against poor reproductive fitness which is usually not a conscious thought or experience on the part of the person. On the other hand, more experiential underlying mechanisms of suicide, such as ongoing psychological pain, have not been explored systematically (Syme et al., 2016, p. 181). This leads us to a discussion on other evolutionary explanations of suicide.

Turning to the bargaining model of SIB, there is existing evidence of threats of self-killing getting converted into suicide attempts and resulting in completed suicides (Whitlock and Knox, 2007, p. 636–637). As mentioned previously in the text, this model has been borrowed from the bargaining model of depression, which posits that severe and disabling symptoms of depression may gain social support and other desirable favors for the person (see Hagen, 2003 for the theory). Along similar lines, the model holds that SIB in persons may also serve to gain attention of significant peers and gain other desirable social effects (Syme et al., 2016, p. 181). The theory views completed suicides as often an accidental and unintentional act. Thus, it might be logically deduced that as long as suicidal threats and SIB serve the purpose of gathering desired social outcomes, the person might not resort to killing self. However, this might not always hold true as there are several factors that determine the translation of SIB either into subsequent suicide or into accidental death. Death by suicide and accidental death following SIB are two distinctive acts that have been classified into separate categories and studied as two discrete populations (Bergen et al., 2012, p. 727–741). Factors that indicate risk of subsequent suicide but not of accidental death in the vulnerable SIB population are incidents of past self-harm such as self-cutting, self-poisoning, and self-injury, and associated psychological underpinnings facilitating self-harm (John, 2012, p. 101). Similarly, factors that signal risk for accidental death (and not for suicide) following self-harm include unemployment, illness, disability, and substance abuse (John, 2012, p. 101). The preceding findings and prior research evidence point toward shortcomings of the bargaining model of suicide which conceptualizes suicide as an unintentional outcome of SIB. Also, similar to the inclusive fitness theory of suicide, the critique of the bargaining model lies in its discussion of ultimate explanations concerning fitness consequences of SIB and why it is selected and not on proximate explanations of the mechanisms that underpin the behavior (Syme et al., 2016, p. 181). As observed earlier, to fully understand any behavior, both proximate and ultimate factors need to be understood (Scott-Phillips et al., 2011, p. 38).

This brings us to one of the latest evolutionary contentions on suicide, the “Pain and Brain” model. This relatively recent evolutionary understanding has addressed both ultimate and proximate factors while explaining human suicide. While stressing the importance of human cognition in the ability to deliberately kill oneself, the model also elaborates the role of psychological pain and an array of psychological factors called “fenders” (first-line evolved psychological mechanisms that regulate the experience of emotional pain and/or keep the option of suicide cognitively unavailable) and “keepers” (last-line evolved psychological mechanisms, like numbing of intense pain or a temporary degradation in planning abilities, that work to prevent intentional self-killing) which determine the probability of self-destructive acts (Soper, 2018). The role of evolved human cognition in conceiving self-killing as an option has also been highlighted by other evolutionary theorists (Humphrey, 2018). According to this model, suicide might not be considered so much as evolutionarily adaptive as it is psychologically adaptive and the theory views suicide as a maladaptive by-product of other adaptive factors: namely, pain and the developed human brain (Soper, 2018, p. 125–244). The “pain and brain” model is in many ways a wholistic approach toward explaining suicide. The theory provides certain intriguing claims, like the evolution of common mental disorders (CMDs) is to prevent self-destructive behaviors such as suicide. According to the author, CMDs, such as depression, substance abuse, or psychosis, involve such degrees of psychological pain and significant loss of functionality that the individual hardly has any ability to contemplate or carry out suicidal acts (Soper, 2018, p. 154). The author provides various arguments to support this hypothesis, such as symptoms of CMDs developing as reactions to emotional pain which in turn would serve as triggers of antisuicide defenses (*Ibid*, p. 157–158). However, this theory has led the present authors to contemplate on the possibility of another evolutionary explanation of the role of emotional pain in suicide. For example, the evolutionarily adaptive vasovagal syncope (or common fainting) in humans involves a drop in blood pressure as a self-preservative mechanism against the experience of emotional pain (Blanc et al., 2015). Can a similar line of reasoning be adopted to understand the role of psychological pain and CMDs in self-preservation of individuals at risk of self-killing? Contentions such as these can be subjected to experimental verification which might eventually contribute to developing preventive measures for individuals experiencing intense psychological pain.

This discussion reinforces the preexisting knowledge that the complex act of suicide in a socially and cognitively advanced species cannot be viewed through an exclusively biological lens. To frame a holistic theory of suicide, both ultimate functions highlighted by evolutionists and proximate mechanisms discussed by psychologists need to be considered in unison. The following section identifies few psychological mechanisms that act proximately and can be integrated into an evolutionary conceptualization to explain suicide. This multifaceted human act calls for interdisciplinary theorizing and this article attempts to link just two of many relevant fields in this area: Evolution and Psychology.

## Linking Evolutionary and Psychological Concepts: A Search for Proximate Explanations

Up until now, we have been proposing the need to identify proximate explanatory factors of suicide and the idea of threading them into an evolutionary framework. This section talks about few possible proximate factors that can be linked with ultimate factors to explain suicide from an evolutionary standpoint. Suicide research points toward an integration of contextual factors (demographics, situational, and behavioral patterns) with proximal processes (personal factors, coping strategies, and emotional states; O’Connor et al., 2015) in building ecologically sound methods of suicide risk assessment (Bronfenbrenner and Morris, 2006). Along these lines, the current paper attempted a brief literature search of studies over the last decade to identify relevant proximal factors predicting a risk of suicide which could be theoretically connected to evolutionary understandings.

We present a brief summary of understandings that emerged from a preliminary literature search of personal factors predicting suicide. The authors identified emotional, cognitive, and behavioral factors mentioned as proximate predictors/indicators of suicide in non-psychiatric samples and then looked for evolutionary explanations for these factors. The purpose is to just begin conceptualizing proximal personal factors of suicide in evolutionary terms rather than systematically reviewing all available data on risk factors of suicide. We hope to convince the scientific audience about the potential of evolutionary psychology in explaining suicide that might culminate in developing interventions that focus on building or re-kindling the basic survival instinct. Table 1 below provides a basic commentary to this end. It is not an exhaustive review of available data. The aim is to lay the groundwork for more systematic and comprehensive review of literature that this area demands. The current article has been drafted as a conceptual analysis and holds promise in being developed into a rigorous and systematic review in the future.

**Table 1:** Summary of Evolutionary explanations for proximate personal factors predicting suicide.

Sr. No.	Methodology	Proximate determinants of suicidal behaviors	Main findings	Reference	Evolutionary interpretations
1.	Prospective study assessing suicide attempters on the HADS, SPS, BHS, Defeat Scale, Entrapment Scale and followed-up after 4years	Sense of entrapment and past suicide attempts (in multivariate analysis)	Feelings of entrapment and past suicidal behaviors can reliably predict future suicide attempts in at-risk individuals	O’Connor, 2003	The social rank theory attributes certain psychopathologies to the evolution of social hierarchies where individual’s perception of success or failure in conflict situations has neurobiological concomitants (Gilbert and Allan, 1998); and, perceiving oneself as having a lower rank as compared to others is associated with feelings of defeat and entrapment which is predictive of SIB (Wetherall et al., 2019). Lowered perception of social rank leading to negative evaluations of self and self-esteem (Kraus and Park, 2014) is a known proximate factor of SIB (O’Connor et al., 2015).
2a.	Systematic review and Meta-analysis of publications reporting D-IAT scores and suicide attempts	Implicit identification with death	The D-IAT scores of an individual may have a role in assessing suicide risk among other determining factors which need to be considered during clinical decision-making.	Sohn et al., 2021	Biased attention to threatening stimuli appears early in life and has possible evolutionary roots (Morales et al., 2016). There is an evolved need to seek life and avoid death (Cha et al., 2018). Associating oneself with death and focusing on suicide-related stimuli might signal the presence of certain dysfunctional evolutionary schemata where the threat to life is stemming from the person him/herself in the form of his/her suicidality (Chiurliza et al., 2018).
b.	Prospective study assessing response of suicide attempters to the modified EST and followed for 6months	Biased attention toward suicide-related words compared to neutral words	Individuals with suicide attempts show an attentional bias toward suicide-related words, predicting consequent suicide attempts over the next 6months, making attentional bias to suicide-related stimuli a possible behavioral marker of suicide risk.	Cha et al., 2010	
c.	Longitudinal study assessing response of college students on the modified EST	Biased attention to suicide-related cues	EST latencies for suicide-related stimuli and past suicidal attempts were significant predictors of suicide risk at follow-up.	Chung and Jeglic, 2017	
3.	Exploratory study assessing recent suicide attempters on the Suicide Intent Scale SIS, Lethality Scale, NDSA, and structured clinical interviews	Impulsivity	Impulsive suicidal attempts are often as lethal as premediated attempts. Individuals with lower levels of depression or hopelessness are often at risk of impulsive suicidal attempts and deserve specific clinical attention.	Spokas et al., 2012	The impulsivity-SIB relationship indicates impulsive aggression directed at self and others in response to acute stress (Mann and Currier, 2009). Evolutionary biologists have identified lower propensities of impulsive aggression in humans as compared to other primates (e.g., chimpanzees). Thus, the presence of impulsive aggression toward self might be a derangement of evolved human tendencies (Chiurliza et al., 2018).
4a.	Longitudinal study of assessing community adults on an adapted version of the CRI at baseline and on the HDL form, negative life events, alcohol use and suicidal ideation at 13years follow-up	Avoidant coping	Use of avoidance coping was positively associated with drinking problems and suicidal ideation at follow-up with increased levels of avoidance coping seen in men and younger adults.	Woodhead et al., 2014	Evolutionary theories of human emotion have explained how negatively valenced stimuli trigger avoidance responses which have proposed evolutionary functions of helping the organism in rapidly evading harmful and threatful stimuli (Schützwohl, 2018). Avoidance of threat often happens *via* an evolved mechanism of disengagement (mental and/or behavioral) which often leads to denial of the problem or feelings of helplessness (Gutiérrez et al., 2007) which are identified precursors of suicidal behaviors (Konkan et al., 2014).
b.	Individuals with suicide attempts and matched healthy controls assessed on the COPE	Maladaptive coping strategies relying more on avoidance and emotional venting	Individuals with suicide attempt showed “lesser active coping,” “lack of planning,” “inability to positively interpret events/situations,” and “indifference to personal growth,” “acceptance,” “emotional impulsivity,” “behavioral disengagement,” “substance abuse” and “avoidance behavior”	Konkan et al., 2014	
5.	Individuals with and without a history of suicidal ideation and attempts were assessed on the ASADI-L, SBQR, BDI-II, BAI, BSS, BAM, ISI, INQ15, ACSS, PPES, BHS, BRFL	Suicidal intent, social disconnection, agitation and other related psychological experiences which together comprise the Acute Suicidal Affective Disturbance (ASAD) construct	Continued experience of ASAD symptoms predicted past suicide attempts more than other suicide risk factors. ASAD symptoms have been validated as proximate risk factors of suicide which deserves particular clinical attention.	Tucker et al., 2016	Overarousal states (e.g., agitation, decreased sleep, cognitive overarousal) seen in humans at risk for suicide appear similar to heightened activity seen in insects just before engaging in self-sacrificial behaviors. Evolutionarily, elevated arousal aides in launching a violent and aggressive attack on predators. In humans, death caused by suicide can be alarming and physically agonizing (Van Orden et al., 2010), as would be any external predator. Hence, elevated levels of agitation and arousal possibly contributes to the individual overcoming his/her innate biological instincts to ultimately engage in a lethal act of self-harm (Chiurliza et al., 2018).

HADS, Hospital Anxiety and Depression Scale; SPS, Suicide Probability Scale; BHS, Beck Hopelessness Scale; D-IAT, Death-Implicit Association Test; EST, Emotional Stroop task; NDSA, Number and Dates of Suicide Attempts; CRI, Coping Responses Inventory; HDL, Health and Daily Living Form; COPE, Coping Orientation to Problems Experienced Inventory; ASADI-L, Acute Suicidal Affective Disturbance Inventory-Lifetime; SBQR, Suicide Behaviors Questionnaire-Revised; BDI-II, Beck Depression Inventory-II; BAI, Beck Anxiety Inventory; BSS, Beck Scale for Suicidal Ideation; BAM, Brief Agitation Measure; ISI, Insomnia Severity Inventory; INQ15, Interpersonal Needs Questionnaire-15; ACSS, Acquired Capability for Suicide Scale; PPES, Painful and Provocative Events Scale; BRFL, Brief Reasons for Living inventory.

There is unanimity among researchers regarding the fact that suicide is an outcome of effectual interactive relationship between biological, psychological, and social factors (Van Heeringen, 2001). Conceptually, suicidal behaviors do not always follow a linear progression from ideation to behavior. Instead, suicidal behavior is the cumulative effect of long-term and short-term risk factors which can produce ideations and behaviors all at once or within a short interval (Bloch-Elkouby et al., 2020).

The tabular discussion below views suicide from an evolutionary lens and indicates that there are complex sets of determining factors at play maybe few days or just moments before the act. These risk factors often do not work in isolation. The risk-protective factor model of suicidal behavior supports this notion that suicidal behaviors are integrated outcomes of various risk factors, and lack of protective factors specific to a given time, situation, and individual (Sánchez, 2001).

To think of ultimate and proximate causation as isolated evolutionary pathways to explaining suicide would be erroneous. These factors are more complimentary in nature, and they act together to determine the likelihood that a particular person, at a specific point of time, in his/her unique contextual settings would consider ending his/her life as both a possibility and a necessity. Evolutionary understandings could throw some light on what is expected to come out of the decision to end one’s own life and why that decision was preferred over the decision to continue living that life. It is a complex calculation which often happens in the blink of an eye, while at other times, it painstakingly accumulates over time. No matter what the progression may look like, the cognitive, behavioral, and emotional processes surrounding a person’s fatal decision might be reflective of conscious or unconscious beliefs about two things: What difference would their absence make to the world they leave behind and the life that they decide to end. Evolutionary processes can not only attempt to answer these questions but also provide an individual with suicidal ideations or intentions an insight into the most fundamental purpose of his/her own life and death.

## Conclusion

Healthcare advocates around the globe are encouraging individuals to participate in non-judgmental dialogue about suicidal cognitions and behavior. Evolutionarily, it is not maladaptive to be in pain. Psychological pain can serve as a signal that there is an underlying problem which needs to be solved, just like physiological pain signals underlying neural or tissue damage (Walters and Williams, 2019). What motivates an individual to decide that the termination of intense psychological pain lies in ending life entirely is an evolutionary paradox in itself. Can the answer lie in the evolution, not of pain alone, but of hedonism, the ultimate human desire for pleasure and escape from pain? Such an approach could seem like a simple addition to or variation of already known approaches that have focused mainly on pain, or as Shneidman has coined “psychache” (Shneidman, 1993, p. 145).

Theories discussing cognition or consciousness in humans as the reason why suicide is considered an option essentially describe why humans are able to contrive of suicide, like any other planned act that we are capable of. However, what those theories do not seem to explain is what makes suicide a compelling choice to a distressed human being and what has made this act seemingly resistant to evolutionary pressures. Talking about psychological mechanisms, coupled with evolutionary gains to chalk an integrated theory of suicide, is just one way of many other possible paths to understanding human suicide. Other schools of thought, like sociology and neurobiology, can also be linked to the underutilized evolutionary approach (Chiurliza et al., 2018) to build a wholistic understanding of human suicide.

## Implications

Viewing suicide through an evolutionary psychology lens will not only lend toward stimulating systematic studies in scientific circles, but also to developing measures of suicide prevention to save valuable lives. There is a need to make a paradigm shift in treating suicide as a derangement or a disordered condition (Soper, 2019b) to viewing it from the vantage point of evolution as an ever-present danger to human existence (Soper, 2019a). Despite developments in social welfare programs across nations resulting in suicide’s adaptive function being apparently dimmed, suicide still continues to exist (Aubin et al., 2013). This further strengthens the need to look beyond the ultimate function of the act while treatment planning at community and individual levels. While psychologically oriented treatment modalities may focus on correcting distorted ways of thinking as one of its techniques of suicide prevention (Stanley et al., 2009), preventive strategies guided by evolutionary psychology would also attempt to explore complex evolved motivations which an individual may often be unaware of and address it in the context of a challenging and ever-evolving social environment (Graham and Martin, 2012). If we understand and address these underlying mechanisms, we can design treatment modalities which might be effective in deterring certain instances of suicide which have been conceptualized in terms of both ultimate and proximate causations of the act.

## Author Contributions

DC has written the manuscript. RR has provided guidance and direction in writing the manuscript. All authors contributed to the article and approved the submitted version.

## Conflict of Interest

The authors declare that the article was written in the absence of any commercial or financial relationships that could be construed as a potential conflict of interest.

## Publisher’s Note

All claims expressed in this article are solely those of the authors and do not necessarily represent those of their affiliated organizations, or those of the publisher, the editors and the reviewers. Any product that may be evaluated in this article, or claim that may be made by its manufacturer, is not guaranteed or endorsed by the publisher.

## References

[ref2] Aubin H. J. Berlin I. Kornreich C. (2013). The evolutionary puzzle of suicide. Int. J. Environ. Res. Public Health 10, 6873–6886. doi: 10.3390/ijerph10126873, PMID: 24351787 PMC3881146

[ref3] Baechler J. (1975/1979). Les Suicides (Cooper B., Trans.). New York: Basic Books.

[ref4] Baumeister R. F. (1990). Suicide as escape from self. Psychol. Rev. 97, 90–113. doi: 10.1037/0033-295X.97.1.90, PMID: 2408091

[ref5] Beck A. T. Brown G. Berchick R. J. Stewart B. L. Steer R. A. (1990). Relationship between hopelessness and ultimate suicide: A replication with psychiatric outpatients. Am. J. Psychiatr. 147, 190–195. doi: 10.1176/ajp.147.2.190 2278535

[ref6] Beck A. T. Kovacs M. Weissman A. (1975). Hopelessness and suicidal behavior: An overview. JAMA 234, 1146–1149. doi: 10.1001/jama.1975.03260240050026, PMID: 1242427

[ref7] Bergen H. Hawton K. Kapur N. Cooper J. Steeg S. Ness J.. (2012). Shared characteristics of suicides and other unnatural deaths following non-fatal self-harm? A multicentre study of risk factors. Psychol. Med. 42, 727–741. doi: 10.1017/S0033291711001747, PMID: 21910932

[ref8] Blanc J. J. Alboni P. Benditt D. G. (2015). Vasovagal syncope in humans and protective reactions in animals. EP Eur. 17, 345–349. doi: 10.1093/europace/euu367, PMID: 25662986

[ref9] Bloch-Elkouby S. Gorman B. Lloveras L. Wilkerson T. Schuck A. Barzilay S.. (2020). How do distal and proximal risk factors combine to predict suicidal ideation and behaviors? A prospective study of the narrative crisis model of suicide. J. Affect. Disord. 277, 914–926. doi: 10.1016/j.jad.2020.08.088, PMID: 33065834

[ref11] Bronfenbrenner U. Morris P. A. (2006). “The bioecological model of human development,” in Theoretical Models of Human Development. Volume 1 of Handbook of Child Psychology. 6th *Edn*. eds. Damon W. Lerner R. M. (Hoboken, NJ: Wiley).

[ref10] Brown R. M. Brown S. L. Johnson A. Olsen B. Melver K. Sullivan M. (2009). Empirical support for an evolutionary model of self-destructive motivation. Suicide Life Threat. Behav. 39, 1–12. doi: 10.1521/suli.2009.39.1.1, PMID: 19298145

[ref12] Cha C. B. Najmi S. Park J. M. Finn C. T. Nock M. K. (2010). Attentional bias toward suicide-related stimuli predicts suicidal behavior. J. Abnorm. Psychol. 119, 616–622. doi: 10.1037/a0019710, PMID: 20677851 PMC2994414

[ref13] Cha C. B. O’Connor R. C. Kirtley O. Cleare S. Wetherall K. Eschle S.. (2018). Testing mood-activated psychological markers for suicidal ideation. J. Abnorm. Psychol. 127, 448–457. doi: 10.1037/abn0000358, PMID: 29927267

[ref14] Chiurliza B. Rogers M. L. Schneider M. E. Chu C. Joiner T. E. (2018). Evolutionary processes in suicide. Curr. Opin. Psychol. 22, 84–88. doi: 10.1016/j.copsyc.2017.08.038, PMID: 28961457 PMC5851811

[ref15] Chung Y. Jeglic E. L. (2017). Detecting suicide risk among college students: A test of the predictive validity of the modified emotional Stroop task. Suicide Life Threat. Behav. 47, 398–409. doi: 10.1111/sltb.12287, PMID: 27658610

[ref16] Ciuhodaru T. Iorga M. Romedea S. N. (2012). Risk factors for iterative non-lethal self-injury. Procedia Soc. Behav. Sci. 33, 766–770. doi: 10.1016/j.sbspro.2012.01.225

[ref17] Conwell Y. Van Orden K. Caine E. D. (2011). Suicide in older adults. Psychiatr. Clin. 34, 451–468. doi: 10.1016/j.psc.2011.02.002, PMID: 21536168 PMC3107573

[ref19] Darwin C. (1909). The Origin of Species. New York: PF Collier and son.

[ref21] deCatanzaro D. (1980). Human suicide: A biological perspective. Behav. Brain Sci. 3, 265–272. doi: 10.1017/S0140525X0000474X

[ref22] deCatanzaro D. (1981). Suicide and self-damaging behavior: A sociobiological perspective. *Vol*. 28. New York: Academic Press.

[ref23] deCatanzaro D. (1984). Suicidal ideation and the residual capacity to promote inclusive fitness: A survey. Suicide Life Threat. Behav. 14, 75–87. doi: 10.1111/j.1943-278x.1984.tb00339.x, PMID: 6515694

[ref24] deCatanzaro D. (1986). A mathematical model of evolutionary pressures regulating self-preservation and self-destruction. Suicide Life Threat. Behav. 16, 166–181. doi: 10.1111/j.1943-278x.1986.tb00350.x, PMID: 3750363

[ref25] deCatanzaro D. (1991). Evolutionary limits to self-preservation. Ethol. Sociobiol. 12, 13–28. doi: 10.1016/0162-3095(91)90010-N

[ref26] deCatanzaro D. (1995). Reproductive status, family interactions, and suicidal ideation: surveys of the general public and high-risk groups. Ethol. Sociobiol. 16, 385–394. doi: 10.1016/0162-3095(95)00055-0

[ref20] De Leo D. Goodfellow B. Silverman M. Berman A. Mann J. Arensman E.. (2021). International study of definitions of English-language terms for suicidal behaviours: a survey exploring preferred terminology. BMJ Open 11:e043409. doi: 10.1136/bmjopen-2020-043409, PMID: 33563622 PMC7875264

[ref27] Durkheim E. (1897). Le suicide: étude de sociologie. Paris: F. Alcan Press.

[ref28] Durkheim E. (1958). Suicide; a Study in Sociology. trans. Spaulding J. A. Simpson G. (Glencoe, IL: The Free Press).

[ref29] Eisenberger N. I. (2012). The neural bases of social pain: evidence for shared representations with physical pain. Psychosom. Med. 74, 126–135. doi: 10.1097/PSY.0b013e3182464dd1, PMID: 22286852 PMC3273616

[ref30] Geulayov G. Casey D. Bale L. Brand F. Clements C. Farooq B.. (2019). Suicide following presentation to hospital for non-fatal self-harm in the multicentre study of self-harm: a long-term follow-up study. Lancet Psychiatry 6, 1021–1030. doi: 10.1016/S2215-0366(19)30402-X, PMID: 31706930

[ref31] Gilbert P. Allan S. (1998). The role of defeat and entrapment (arrested flight) in depression: an exploration of an evolutionary view. Psychol. Med. 28, 585–598. doi: 10.1017/S0033291798006710, PMID: 9626715

[ref32] Graham R. G. Martin G. I. (2012). Health behavior: a Darwinian reconceptualization. Am. J. Prev. Med. 43, 451–455. doi: 10.1016/j.amepre.2012.06.016, PMID: 22992365

[ref33] Grandclerc S. De Labrouhe D. Spodenkiewicz M. Lachal J. Moro M. R. (2016). Relations between nonsuicidal self-injury and suicidal behavior in adolescence: a systematic review. PLoS One 11:e0153760. doi: 10.1371/journal.pone.0153760, PMID: 27089157 PMC4835048

[ref35] Gutiérrez F. Peri J. M. Torres X. Caseras X. Valdés M. (2007). Three dimensions of coping and a look at their evolutionary origin. J. Res. Pers. 41, 1032–1053. doi: 10.1016/j.jrp.2007.01.006

[ref36] Hagen E. H. (1999). The functions of postpartum depression. Evol. Hum. Behav. 20, 325–359. doi: 10.1016/S1090-5138(99)00016-1

[ref37] Hagen E. H. (2003). “The bargaining model of depression,” in Genetic and Cultural Evolution of Cooperation. ed. Hammerstein P. (Cambridge: MIT Press), 95–123.

[ref38] Hamilton W. D. (1964). The genetical evolution of social behaviour. I. J. Theor. Biol. 7, 1–16. doi: 10.1016/0022-5193(64)90038-4, PMID: 5875341

[ref39] Hamilton W. D. (1970). Selfish and spiteful behaviour in an evolutionary model. Nature 228, 1218–1220. doi: 10.1038/2281218a0, PMID: 4395095

[ref40] Hamilton W. D. (1971). “Selection of selfish and altruistic behavior in some extreme models,” in Man and Beast: Comparative Social Behavior. eds. Eisenberg J. F. Dillon W. S. (Washington, DC: Smithsonian Institution Press), 57–91.

[ref42] Harrison K. E. Dombrovski A. Y. Morse J. Q. Houck P. Schlernitzauer M. Reynolds C. F. III. (2010). Alone? Perceived social support and chronic interpersonal difficulties in suicidal elders. Int. Psychogeriatr. 22, 445–454. doi: 10.1017/S1041610209991463, PMID: 20003633 PMC3045785

[ref44] Humphrey N. (2018). The lure of death: suicide and human evolution. Philos. Trans. R. Soc. Lond., B, Biol. Sci. 373:20170269. doi: 10.1098/rstb.2017.0269, PMID: 30012736 PMC6053977

[ref45] Humphreys R. K. Ruxton G. D. (2019). Adaptive suicide: is a kin-selected driver of fatal behaviours likely? Biol. Lett. 15:20180823. doi: 10.1098/rsbl.2018.0823, PMID: 30958139 PMC6405458

[ref47] John A. (2012). Following self-harm, there are shared and differing risk factors for subsequent suicide death or accidental death. Evid.-Based Ment. Health 15:101. doi: 10.1136/eb-2012-100944, PMID: 22949630

[ref48] Joiner T. E. (2005). Why People Die by Suicide. Cambridge, MA: Harvard University Press.

[ref52] Klein C. (2015). What the Body Commands: The Imperative Theory of Pain. Cambridge: MIT Press.

[ref53] Klonsky E. D. May A. M. (2015). The three-step theory (3ST): A new theory of suicide rooted in the “ideation-to-action” framework. Int. J. Cogn. Ther. 8, 114–129. doi: 10.1521/ijct.2015.8.2.114

[ref54] Konkan R. Erkuş G. H. Güçlü O. Şenormanci Ö. Aydin E. Ülgen M. C.. (2014). Coping strategies in patients who had suicide attempts. Nöro Psikiyatr. Arş. 51, 46–51. doi: 10.4274/npa.y6578, PMID: 28360594 PMC5370265

[ref56] Kraus M. W. Park J. W. (2014). The undervalued self: social class and self-evaluation. Front. Psychol. 5:1404. doi: 10.3389/fpsyg.2014.01404, PMID: 25538654 PMC4256993

[ref58] Linehan M. M. (1993). Cognitive-Behavioral Treatment of Borderline Personality Disorder. New York: Guilford Press.

[ref59] Mann J. J. Currier D. (2009). “Biological predictors of suicidal behavior in mood disorders,” in Oxford Textbook of Suicide Prevention: A Global Perspective. eds. Wasserman D. Wasserman C. (Oxford, UK: Oxford University Press), 335–341.

[ref60] Mars B. Heron J. Klonsky E. D. Moran P. O’Connor R. C. Tilling K.. (2019). Predictors of future suicide attempt among adolescents with suicidal thoughts or non-suicidal self-harm: a population-based birth cohort study. Lancet Psychiatry 6, 327–337. doi: 10.1016/S2215-0366(19)30030-6, PMID: 30879972 PMC6494973

[ref62] Morales S. Fu X. Pérez-Edgar K. E. (2016). A developmental neuroscience perspective on affect-biased attention. Dev. Cogn. Neurosci. 21, 26–41. doi: 10.1016/j.dcn.2016.08.001, PMID: 27606972 PMC5067218

[ref63] Nesse R. M. (2000). Is depression an adaptation? Arch. Gen. Psychiatry 57, 14–20. doi: 10.1001/archpsyc.57.1.14, PMID: 10632228

[ref65] O’Connor R. C. (2003). Suicidal behavior as a cry of pain: test of a psychological model. Arch. Suicide Res. 7, 297–308. doi: 10.1080/713848941

[ref64] O’Connor R. C. (2011). The Integrated Motivational-Volitional Model of Suicidal Behavior. Crisis 32, 295–298. doi: 10.1027/0227-5910/a000120, PMID: 21945841

[ref66] O’Connor M. Dooley B. Fitzgerald A. (2015). Constructing the suicide risk index (SRI): does it work in predicting suicidal behavior in young adults mediated by proximal factors? Arch. Suicide Res. 19, 1–16. doi: 10.1080/13811118.2014.915775, PMID: 25058873

[ref67] O’Connor R. C. Kirtley O. J. (2018). The integrated motivational–volitional model of suicidal behaviour. Philos. Trans. R. Soc. Lond., B, Biol. Sci. 373:20170268. doi: 10.1098/rstb.2017.0268, PMID: 30012735 PMC6053985

[ref68] Perry S. (2014). Every Cradle Is a Grave: Rethinking the Ethics of Birth and Suicide. Charleston, WV: Nine-Banded Books.

[ref69] Poulton E. B. (1890). The Colours of Animals: Their Meaning and Use, Especially Considered in the Case of Insects: New York: D. Appleton.

[ref70] Quarshie E. N. Waterman M. G. House A. O. (2020). Self-harm with suicidal and non-suicidal intent in young people in sub-Saharan Africa: a systematic review. BMC Psychiatry 20:234. doi: 10.1186/s12888-020-02587-z, PMID: 32408896 PMC7222461

[ref72] Roberts M. Lamont E. (2014). Suicide: an existentialist reconceptualization. J. Psychiatr. Ment. Health Nurs. 21, 873–878. doi: 10.1111/jpm.12155, PMID: 24796698

[ref73] Rowe C. Guilford T. (2000). Aposematism: to be red or dead. Trends Ecol. Evol. 15, 261–262. doi: 10.1016/S0169-5347(00)01897-8

[ref74] Rudd M. D. (2006). “Fluid Vulnerability Theory: A Cognitive Approach to Understanding the Process of Acute and Chronic Suicide Risk,” in Cognition and Suicide: Theory, Research, and Therapy. ed. Ellis T. E. (Washington DC: American Psychological Association), 355–368.

[ref75] Sánchez H. G. (2001). Risk factor model for suicide assessment and intervention. Prof. Psychol. Res. Pract. 32, 351–358. doi: 10.1037/0735-7028.32.4.351

[ref76] Schmidt J. O. (2008). “Venoms and toxins in insects,” in Capinera J.L. ed. Encyclopedia of Entomology (Dordrecht: Springer).

[ref77] Schützwohl A. (2018). Approach and avoidance during routine behavior and during surprise in a non-evaluative task: surprise matters and so does the valence of the surprising event. Front. Psychol. 9:826. doi: 10.3389/fpsyg.2018.00826, PMID: 29962978 PMC6013581

[ref78] Scott-Phillips T. C. Dickins T. E. West S. A. (2011). Evolutionary theory and the ultimate–proximate distinction in the human behavioral sciences. Perspect. Psychol. Sci. 6, 38–47. doi: 10.1177/1745691610393528, PMID: 26162114

[ref79] Selby E. A. Joiner T. E. Jr. Ribeiro J. D. (2014). “Comprehensive theories of suicidal behaviors,” in The Oxford Handbook of Suicide and Self-Injury. ed. Nock M. K. (Oxford, England: Oxford University Press), 286–307.

[ref80] Shackleton K. Al Toufailia H. Balfour N. J. Nascimento F. S. Alves D. A. Ratnieks F. L. W. (2015). Appetite for self-destruction: suicidal biting as a nest defense strategy in Trigona stingless bees. Behav. Ecol. Sociobiol. 69, 273–281. doi: 10.1007/s00265-014-1840-6, PMID: 25620834 PMC4293493

[ref81] Shah A. Bhat R. Zarate-Escudero S. DeLeo D. Erlangsen A. (2016). Suicide rates in five-year age-bands after the age of 60 years: the international landscape. Aging Ment. Health 20, 131–138. doi: 10.1080/13607863.2015.1055552, PMID: 26094783

[ref82] Shneidman E. S. (1993). Commentary: suicide as psychache. J. Nerv. Ment. Dis. 181, 145–147. doi: 10.1097/00005053-199303000-00001, PMID: 8445372

[ref83] Shorter J. R. Rueppell O. (2012). A review on self-destructive defense behaviors in social insects. Insect. Soc. 59, 1–10. doi: 10.1007/s00040-011-0210-x

[ref84] Sohn M. N. McMorris C. A. Bray S. McGirr A. (2021). The death-implicit association test and suicide attempts: a systematic review and meta-analysis of discriminative and prospective utility. Psychol. Med. 51, 1789–1798. doi: 10.1017/S0033291721002117, PMID: 34030752

[ref85] Soper C. (2018). The Evolution of Suicide. Switzerland: Springer.

[ref86] Soper C. (2019a). Adaptation to the suicidal niche. Evolutionary. Psychol. Sci. 5:10.13140/RG.2.2.26795.08487, 454–471.

[ref87] Soper C. (2019b). “Beyond the Search for Suigiston: How Evolution Offers Oxygen for Suicidology,” in Evolutionary Perspectives on Death eds. Shackelford T. K. Zeigler-Hill V. (Switzerland: Springer), 37–61.

[ref88] Spokas M. Wenzel A. Brown G. K. Beck A. T. (2012). Characteristics of individuals who make impulsive suicide attempts. J. Affect. Disord. 136, 1121–1125. doi: 10.1016/j.jad.2011.10.034, PMID: 22119087 PMC3927642

[ref91] Stanley B. Brown G. Brent D. A. Wells K. Poling K. Curry J.. (2009). Cognitive-behavioral therapy for suicide prevention (CBT-SP): treatment model, feasibility, and acceptability. J. Am. Acad. Child Adolesc. Psychiatry 48, 1005–1013. doi: 10.1097/CHI.0b013e3181b5dbfe 19730273 PMC2888910

[ref89] Stengel E. (1952). Enquiries into Attempted Suicide. Proc. R. Soc. Med. 45, 613–620. doi: 10.1177/003591575204500910, PMID: 13003959 PMC1987521

[ref90] Stengel E. (1956). The social effects of attempted suicide. Can. Med. Assoc. J. 74, 116–120. PMID: 13284662 PMC1826412

[ref92] Syme K. (2017). “Denis Decatanzaro,” in Encyclopedia of Evolutionary Psychological Science. eds. Shackelford T. K. Weekes-Shackelford V. A. (Cham: Springer International Publishing), 1–2.

[ref93] Syme K. (2019). “Bargaining model of suicidal behavior,” in Encyclopedia of Evolutionary Psychological Science. eds. Shackelford T. K. Weekes-Shackelford V. A. (Cham: Springer International Publishing), 1–3.

[ref94] Syme K. L. Garfield Z. H. Hagen E. H. (2016). Testing the bargaining vs. inclusive fitness models of suicidal behavior against the ethnographic record. Evol. Hum. Behav. 37, 179–192. doi: 10.1016/j.evolhumbehav.2015.10.005

[ref95] Szentes B. Thomas C. D. (2013). An evolutionary theory of suicide. Games 4, 426–436. doi: 10.3390/g4030426

[ref96] Tanaka M. Kinney D. K. (2011). An evolutionary hypothesis of suicide: why it could be biologically adaptive and is so prevalent in certain occupations. Psychol. Rep. 108, 977–992. doi: 10.2466/02.12.17.PR0.108.3.977-992, PMID: 21879643

[ref97] Trail D. R. S. (1980). Behavioral interactions between parasites and hosts: host suicide and the evolution of complex life cycles. Am. Nat. 116, 77–91. doi: 10.1086/283612

[ref98] Tucker R. P. Michaels M. S. Rogers M. L. Wingate L. R. Joiner T. E. Jr. (2016). Construct validity of a proposed new diagnostic entity: acute suicidal affective disturbance (ASAD). J. Affect. Disord. 189, 365–378. doi: 10.1016/j.jad.2015.07.049, PMID: 26476421

[ref99] Van Heeringen K. (2001). “The suicidal process and related concepts,” in Understanding Suicidal Behaviour. ed. Van Heeringen K. (Chichester: John Wiley & Sons Ltd.), 136–159.

[ref100] Van Orden K. A. Witte T. K. Cukrowicz K. C. Braithwaite S. R. Selby E. A. Joiner T. E. Jr. (2010). The interpersonal theory of suicide. Psychol. Rev. 117, 575–600. doi: 10.1037/a0018697, PMID: 20438238 PMC3130348

[ref101] Wallace A. (1867). Proceedings of the Entomological Society of London; March 04, 1867.

[ref102] Walters E. T. Williams A. C. D. C. (2019). Evolution of Mechanisms and Behaviour Important for Pain. Philos. Trans. R. Soc. Lond. Ser. B Biol. Sci. 374:20190275. doi: 10.1098/rstb.2019.0275, PMID: 31544614 PMC6790384

[ref103] Wetherall K. Robb K. A. O’Connor R. C. (2019). Social rank theory of depression: A systematic review of self-perceptions of social rank and their relationship with depressive symptoms and suicide risk. J. Affect. Disord. 246, 300–319. doi: 10.1016/j.jad.2018.12.045, PMID: 30594043

[ref104] Whitlock J. Knox K. L. (2007). The relationship between self-injurious behavior and suicide in a young adult population. Arch. Pediatr. Adolesc. Med. 161, 634–640. doi: 10.1001/archpedi.161.7.634, PMID: 17606825

[ref105] Wiley J. C. (2020). Psychological Aposematism: An evolutionary analysis of suicide. Biol. Theory 15, 226–238. doi: 10.1007/s13752-020-00353-8

[ref106] Woodhead E. L. Cronkite R. C. Moos R. H. Timko C. (2014). Coping strategies predictive of adverse outcomes among community adults. J. Clin. Psychol. 70, 1183–1195. doi: 10.1002/jclp.21924, PMID: 23629952

[ref108] World Health Organization (2014). Preventing suicide: A global imperative. World Health Organization. Available at: https://www.who.int/publications/i/item/9789241564779

[ref107] World Health Organization (2018). Crude Suicide Rates. Available at: https://apps.who.int/gho/data/node.sdg.3-4-viz-2?lang=en (Accessed February 8, 2021).

